# Vitrectomized versus non-vitrectomized eyes in diabetic macular edema response to ranibizumab—retinal layers thickness as prognostic biomarkers

**DOI:** 10.1038/s41598-021-02532-4

**Published:** 2021-11-29

**Authors:** Bernardete Pessoa, João Leite, João Heitor, João Coelho, Sérgio Monteiro, Constança Coelho, João Figueira, Angelina Meireles, João Nuno Melo-Beirão

**Affiliations:** 1 Departamento de Oftalmologia, Hospital de Santo António, Centro Hospitalar Universitário do Porto, Largo Prof. Abel Salazar—Edifício Neoclássico, 4099-001 Porto, Portugal; 2 Instituto de Ciências Biomédicas Abel Salazar (ICBAS), Universidade do Porto, Porto, Portugal; 3 Departamento de Oftalmologia, Hospital de Santa Maria Maior de Barcelos, Barcelos, Portugal; 4 Instituto de Saúde Ambiental, Faculdade de Medicina, Universidade de Lisboa, Lisbon, Portugal; 5 Centro Hospitalar e Universitário de Coimbra, Coimbra, Portugal; 6 Faculty of Medicine, University of Coimbra, Coimbra, Portugal; 7 Association for Innovation and Biomedical Research On Light and Image, Coimbra, Portugal

**Keywords:** Medical research, Biomarkers

## Abstract

To evaluate the role of the vitreous in the management of diabetic macular edema with ranibizumab intravitreal injections in a pro re nata regimen. Prospective study of 50 consecutive eyes with diabetic macular edema treated with ranibizumab and 12 months of follow-up. Primary endpoint: to assess differences between non-vitrectomized and vitrectomized eyes in the number injections needed to control the edema. Secondary endpoints: comparison of groups regarding best corrected visual acuity, central foveal thickness and thickness of seven retinal layers. 46 eyes from 38 patients, 10 vitrectomized and 36 non-vitrectomized, completed the follow-up. At month 12, the two groups achieved an equivalent anatomical outcome and needed a similar number of ranibizumab intravitreal injections. In vitrectomized eyes final visual acuity was worse when baseline retinal nerve fiber layers in the central foveal subfield were thicker, showing a strong correlation (r = − 0.942, *p* < 0.001). A similar, albeit moderate correlation was observed in non-vitrectomized eyes (r = − 0.504, *p* = 0.002). A decrease of retinal nerve fiber layers inner ring thickness was correlated with a better final visual acuity only in vitrectomized eyes (r = 0.734, *p* = 0.016). The effect of diabetic macular edema seems to be worse in vitrectomized eyes, with a thinner inner retina reservoir.

Clinicaltrials.govNCT04387604.

## Introduction

Diabetic retinopathy (DR) is one of the most important diabetes mellitus microvascular complications and diabetic macular edema (DME) is the main responsible for the vision loss related to DR^1^. The International Council of Ophthalmology and EURETINA Guidelines recommend anti-vascular endothelial growth factors (anti-VEGF) agents as first-line therapy for treating central DME^2^. Among the approved anti-VEGF products, ranibizumab (RBZ) is the one with more safety and efficacy data in the long term^3–8^.

DME pathogenesis is multifactorial and the posterior cortical vitreous seems to play a major role in its development, via several mechanical and physiological mechanisms that lead to increased vascular permeability^1,9–12^. Vitrectomy has been proven to be effective in the resolution of DME, through the removal of growth factors and cytokines in a background of an ischemic retina and inflammatory response, particularly when a tractional cause is involved in its pathogenesis^13,14^. Although DME recurrence post-vitrectomy has been reported in a low percentage of cases (10.9%)^15^, this type of edemas is considered more difficult to treat particularly with intravitreal (IV) anti-VEGF agents since those eyes have a more rapid clearance of drugs than non-vitrectomized eyes^16–19^. There is a lack of long term, prospective, comparative studies addressing vitrectomized and non-vitrectomized eyes with DME and otherwise comparable characteristics. There is some evidence that RBZ is also an effective treatment for DME in vitrectomized eyes although the functional and anatomical efficacy seems to be achieved slower, with the need of a higher number of injections at least during the first 12 months of treatment^1,20,21^.

Neurodegeneration is an early event in DR, documented through the thinning of the inner retina due to ganglion cell apoptosis, reflected also in retinal nerve fiber layers (RNFL), the axons derived from ganglion cells^22,23^. It has already been described that inner and outer temporal ganglion cells complex layer (GCL) thickness is decreased in vitrectomized eyes^24^.

The total retinal thickness in DME seen on OCT may represent edematous or degenerative changes. The effect and negative impact of DME in inner retinal layers and of the latter on functional outcomes has been objectively demonstrated, with the achievement of a positive correlation of inner retinal layers thickness reduction, particularly in the nasal quadrant, and visual gain, both with RBZ and triamcinolone applied to DME treatment^25^. As opposed to non-vitrectomized eyes, the influence of DME in eyes without vitreous is much less explored.

The purpose of this study was to deepen the knowledge of the real effect of the vitreous status in the management of DME with RBZ IV injections, a first line treatment approach for DME. A comprehensive analyses of the different retinal layers thickness before and after treatment, and their influence on functional and anatomical outcomes, was assessed.

## Results

### Demographic and clinical baseline data

From the 50 eyes enrolled in the study, 46 eyes of 38 patients, 10 vitrectomized and 36 non-vitrectomized, completed the entire follow-up. There were no differences between any of the analysed demographic or clinical parameters at baseline—Table 1.

**Table 1:** Baseline characteristics.

Parameter	Group 1 [n = 36]	Group 2 [n = 10]	*p* value
Age, mean [95% CI]	66.45 [62.87–70.02]	67.22 [60.34–74.11]	0.973
Gender, female	55.6%	41.4%	0.703
DM duration (years), mean [95% CI]	17.00 [13.90–20.10]	22.56 [16.26–28.85]	0.068
DME duration (months), mean [95% CI]	28.08 [19.62–36.55]	30.60 [6.39–54.81]	0.803
Type 2 DM	100%	100%	1.000
Macular LASER	80%	63.9%	0.460
PRP LASER	80%	58.3%	0.282
Naive IV	20%	33.3%	0.699
Number previous IV treatments, mean [95% CI]	5.31 [3.10–7.53]	2.50 [(− 0.84)–5.84]	0.179
HbA1c, mean [95% CI]	7.45 [7.12–7.78]	7.59 [6.76–8.42]	0.867
Hemoglobin, mean [95% CI]	12.98 [ 12.29–13.66]	12.91 [11.49–14.33]	0.946
Microalbuminuria, mean [95% CI]	299.14 [20.24–578.03]	117.74 [(− 35.8)–271.27]	0.647
BMI, mean [95% CI]	28.35 [26.96–29.74]	28.17 [24.99–31.35]	1.000
SBP, mean [95% CI]	137.6 [132.5–142.7]	138.5 [127.1–149.9]	0.697
DBP, mean [95% CI]	75.9 [73.2–78.7]	74.3 [65.6–83.0]	0.600

Group 1 = non vitrectomized; Group 2 = vitrectomized; 95% CI = 95% Confidence interval; DM = Diabetes mellitus; DME = diabetic macular edema; macular LASER = Prior focal-grid photocoagulation treatment in the study; PRP LASER = Prior panretinal photocoagulation treatment in the study eye; IV = intravitreal, naïve IV = no prior anti-VEGF intravitreal treatment in study eye; BMI = body mass index, SBP = systolic blood pressure, DBP = diastolic blood pressure.

At baseline, group 2 had thinner inner retinal layers, particularly the GCL layer, with a mean thickness of 35.5 μm (26–43, 95% CI 31.2–39.7) compared to 41.3 μm (32–51, 95% CI 39.8–42.8) in group 1 (*p* = 0.011). A more expressive thinner GCL in group 2, compared with group 1, was observed in inner and outer temporal ETDRS subfields and also in inner ring ETDRS subfields (*p* < 0.001 and *p* = 0.002, respectively)—Table 2.

**Table 2:** Baseline thickness of retinal layers with significant difference between groups.

Parameter	Group 1 [n = 36]	Group 2 [n = 10]	*p* value
GCL int baseline, mean [95% CI]	49.8 [48.2–51.4]	41.8 [35.9–47.6]	**0.002**
IPL ext baseline, mean [95% CI]	31.2 [29.9–32.5]	28.3 [25.4–31.2]	**0.047**
GCL global baseline, mean [95% CI]	41.3 [39.8–42.8]	35.5 [31.2–39.7]	**0.011**
GCL temp baseline, mean [95% CI]	41.1 [38.8–43.3]	32.4 [27.4–37.4]	** < 0.001**
GCL sup baseline, mean [95% CI]	43.0 [41.4–44.7]	37.0 [31.5–42.5]	**0.010**

Group 1 = non vitrectomized; Group 2 = vitrectomized; GCL = Ganglion cell layer; IPL = inner plexiform layer; int = inner ring ETDRS subfields; ext = outer ring ETDRS subfields; global = the nine ETDRS subfields; temp = inner and outer temporal ETDRS subfields; sup = inner and outer superior ETDRS subfields. 95% CI = 95% Confidence interval.

Only statistically significant differences are reported.

All significant values are represented in bold. A *p* < 0.05 was considered statistically significant.

A percentage of 30.4% of the patients was treatment *naïve* (33% and 20%, *p* = 0.699, in group 2 and group 1, respectively). The causes for vitrectomy were PDR in 7 cases, ERM in one case (in these 8 cases ILM was peeled without indocyanine green staining), one vitreous hemorrhage with no PDR and one retinal detachment.

### Number of RBZ IV injections needed to control DME

The mean number of RBZ IV injections needed to control DME was 7.86 (95% CI 5.39–10.33) in vitrectomized eyes and 7.72 (95% CI 6.71–8.74) in non-vitrectomized eyes (*p* = 0.815). Although the number of RBZ IV was similar in both groups, there was an overall association between DME resolution and the number of RBZ IV injections, favoring a lower number of injections (*p* = 0.002). Overall BCVA at month 12 showed a positive moderate correlation with the number of RBZ IV injections (r = 0.562, *p* < 0.001).

### BCVA and CFT evolution at baseline and 12 months follow-up

**Figure 1:**
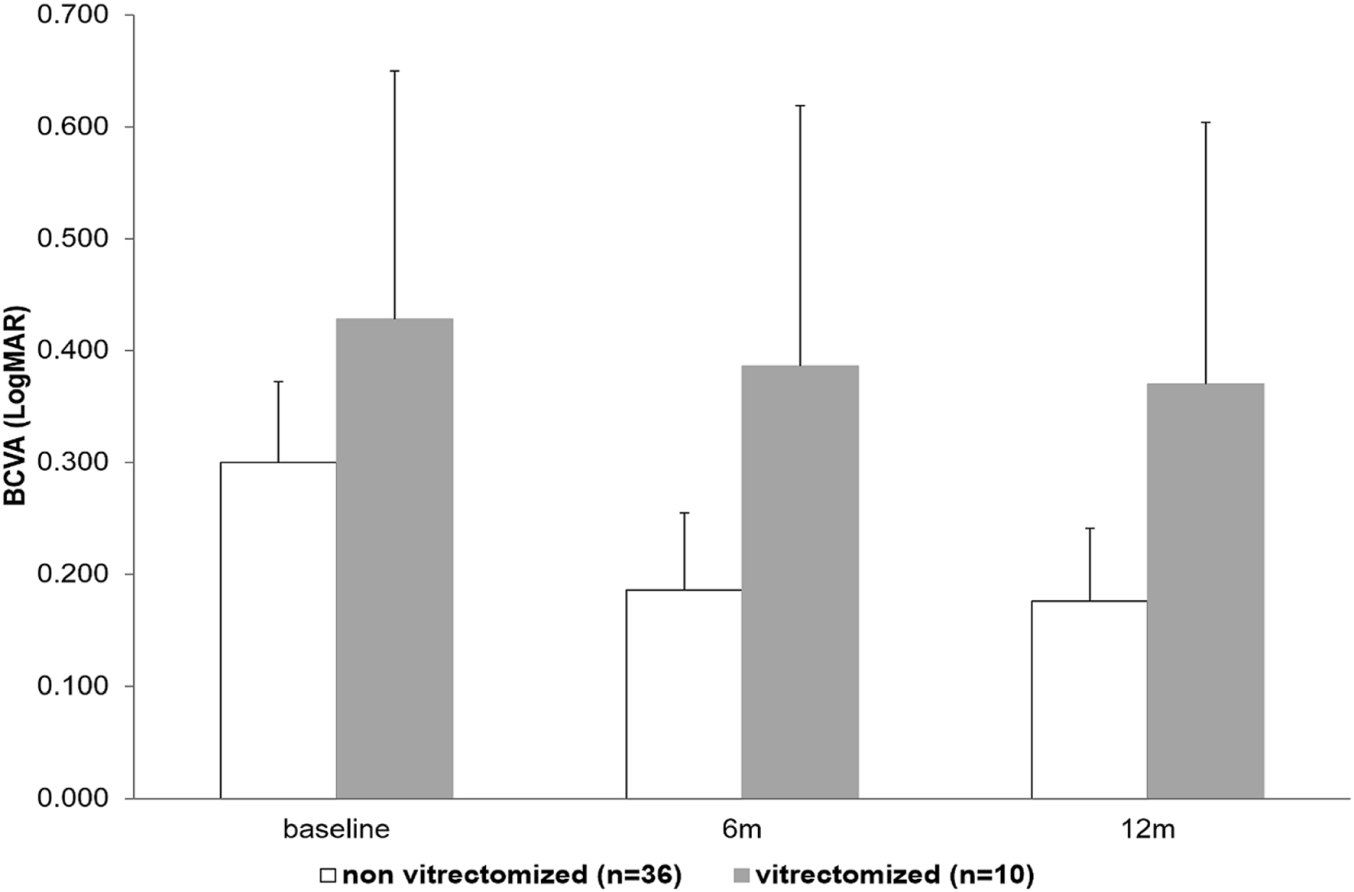
BCVA evolution from baseline to the end of follow-up. Mean ± 95% CI. *p* = ns at each time point using the Mann–Whitney U test.

**Figure 2:**
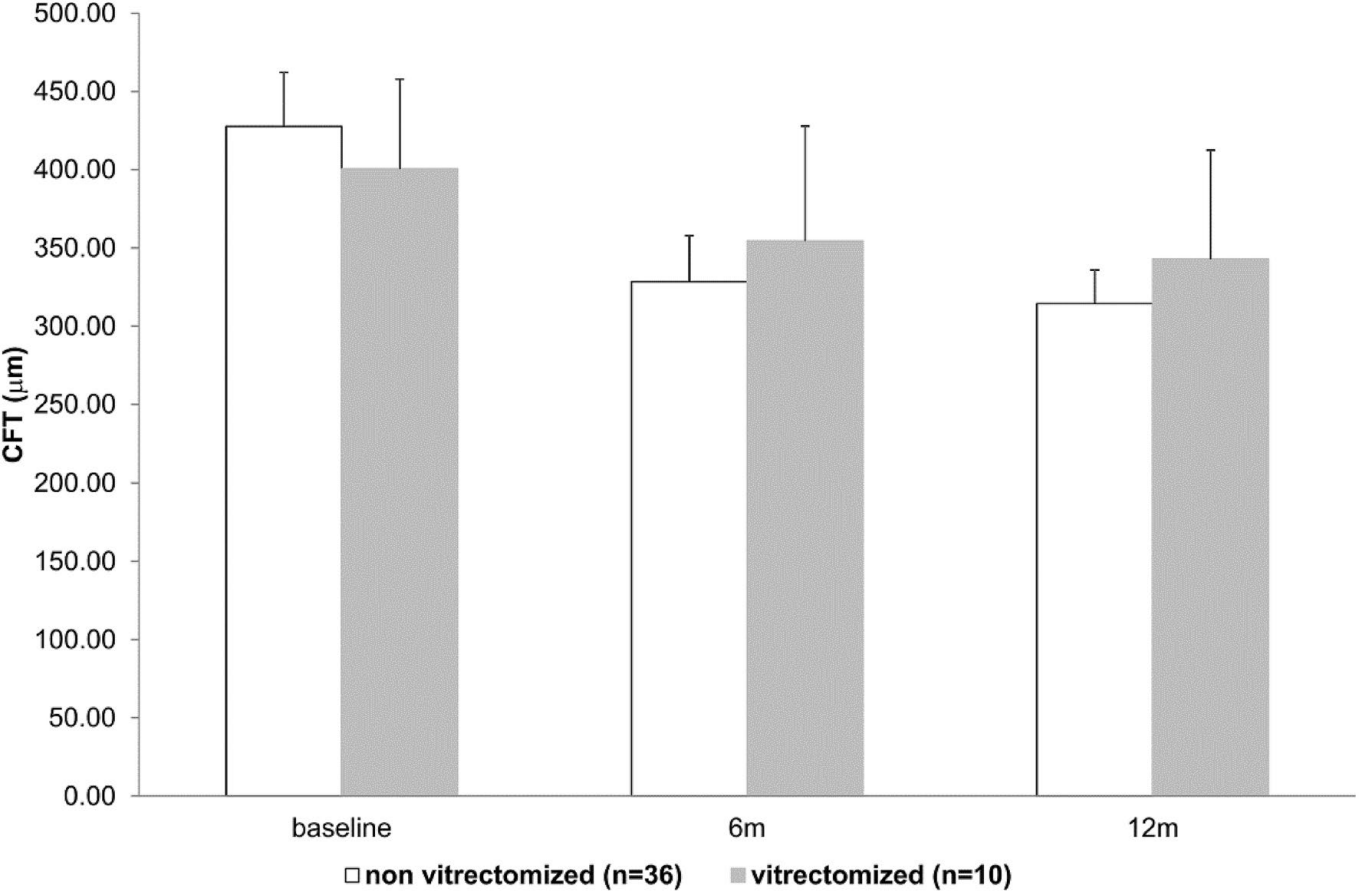
CFT evolution from baseline to the end of follow-up. Mean ± 95% CI. *p* = ns at each time point using the Mann–Whitney U test.

There were no differences between groups regarding BCVA and CFT evolution from baseline to the end of follow-up (Figs. 1, 2, respectively). Nevertheless, at month 12, there were more eyes with < 70 ETDRS letters in group 2 compared to group 1 (50% vs 13.9%, *p* = 0.027).

### Type of responders

There were no differences between groups 1 and 2 regarding the type of responder, good-earlier responders versus partial/non-responders (*p* = 1.000). Overall 33% of patients were good-earlier responders and 69% of good-earlier responders had a baseline CFT < 400 µm.

When analyzing good-earlier responders versus partial/non-responders, good responders were significantly associated with a lower INL thickness at baseline and at the end of follow-up, especially in the inner ring of ETDRS subfield (*p* = 0.002 and *p* = 0.004, respectively). There were no differences between responder groups regarding rescue LASER approach (*p* = 0.225).

### Analysis of the thickness of the seven retinal layers

A statistically significant difference between groups was observed in differences from 12 months follow-up to baseline only for INL and, to a lesser extent, for GCL layers—Table 3.

**Table 3:** Differences from 12 months follow-up to baseline of the different ETDRS subfields.

Parameter	Group 1 [n = 36]	Group 2 [n = 10]	*p* value
Dif INL ext, mean [95% CI]	− 0.5 [(− 1.5)–1.4]	− 1.9 [(− 3.2)–(− 0.6)]	**0.031**
Dif GCL M, mean [95% CI]	− 0.1 [(− 1.9)–1.7]	− 2.5 [(− 3.8)–(− 1.2)]	**0.033**
Dif INL M, mean [95% CI]	− 1.4 [(− 2.6)–(− 0.2)]	− 4.4 [(− 5.7)–(− 3.2)]	**0.007**

Group 1 = non vitrectomized; Group 2 = vitrectomized; GCL = Ganglion cell layer; INL = inner nuclear layer; ext = outer ring ETDRS subfields; M = the nine ETDRS subfields; 95% CI = 95% Confidence interval.

Only statistically significant differences are reported.

All significant values are represented in bold. A *p* < 0.05 was considered statistically significant.

### Correlations between parameters

In vitrectomized eyes final visual acuities were strongly correlated with baseline RNFL and GCL, in the central foveal subfield (r = 0.942 and r = 0.871, respectively, *p* < 0.001). Also, a positive correlation, although moderate, was observed in non-vitrectomized eyes only for RNFL in the central foveal subfield (r = 0.504, *p* = 0.002). A decrease of RNFL inner ring thickness was negatively correlated with the final visual acuity in vitrectomized eyes (r = − 0.734, *p* = 0.016), with no correlation in non-vitrectomized eyes.

### Safety outcomes

Ocular adverse events included a retinal detachment fifteen days after a third IV injection and an iatrogenic cataract at the eighth month of follow-up, both leading to drop-out of the study. No other serious ocular events, such as endophthalmitis, were registered. The two remaining causes of drop-out of the study were due to non-ocular serious adverse events: one acute myocardial infarction (AMI) and one death due to a stroke at the fifth (after four ranibizumab injections) and the fourth month (after three ranibizumab injections) of follow-up, respectively.

## Discussion

This prospective long term study tried to comprehensively analyze vitrectomized and non-vitrectomized eyes in their vitreoretinal anatomical features beyond the simple vitreous’ existence, in the process of DME treatment with a first line treatment approach with RBZ IV injections.

There is some controversy regarding the real effect of the vitreous status and intravitreal anti-VEGF therapy in DME. The majority of studies was performed in animals and showed more rapid clearance rates of bevacizumab, RBZ and triamcinolone acetonide placed inside the vitreous cavity of vitrectomized eyes^16–18^, although others did not^19^. In humans this evidence is even more scarce and there are no studies addressing anti-VEGF drugs^20^. Another variable to consider is the vitreous concentrations of VEGF that are increased and correlated with the severity of macular edema in diabetic patients^21^. Vitrectomy itself may have other positive effects that may offset an eventual inferior half-life of IV agents, through the removal of cytokines, VEGF and advanced glycation end products from the vitreous, facilitating the fluid circulation inside the vitreous cavity, along with the increased macular capillary flow, retinal oxygenation and, not less important, promoting the vitreous macular traction release^13,26^.

According to our study, and in line with this rationale, the number of RBZ IV injections needed to control DME was similar in groups 1 and 2. Another interesting observation was the overall association between DME resolution, a better visual outcome and a lower number of RBZ IV injections, the paradigm of good-earlier responders. These sub-types of patients were not different in groups 1 and 2.

Regarding treatment efficacy between groups this was confirmed in anatomical and functional outcomes, as no differences existed between groups regarding BCVA and CFT evolution during the entire follow-up period.

In vitrectomized eyes, the trend to inferior outcomes and different results, according to different studies, concerning the number of IV anti-VEGF injections, with higher number at least in the first 6 months and first year of treatment^20,21^, and the type of anatomical and functional response (less expressive^20,21^, variable^27^, ineffective^28,29^ or achieved slower^20,21^) can be related with several factors: (1) the different anti-VEGF used^8,30^; (2) the retrospective nature of the studies^20,27,28^; (3) the poor design of studies with different or short term^20,28,29,31^ follow-ups; and (4) the different baseline/demographic characteristics^21^. All of these factors may contribute to the different reported results, in addition to the particular background of a vitrectomized eye.

Regarding the baseline characteristics of our cohort the two groups were very similar. The only significant difference was the expected decrease in inner retinal layers^32^, particularly in the GCL thickness of inner-outer temporal and superior sectors and in inner ring ETDRS subfields in vitrectomized eyes, with ILM peeling performed in 80% of the eyes in our study. A possible explanation for these results could be the preference for ILM removal in temporal superior sectors due both to ease of access and to preserve the nasal part, where the papillomacular bundle is located, leaving the remaining sectors to be removed clockwise with a minimum touch^32^. Although ILM peeling is indicated to prevent re-proliferation of ERM^33^, one retrospective study found that ILM removal was correlated with worse visual outcomes^34^. Furthermore, retina from diabetic patients is particularly susceptible, as neurodegeneration is also an early event in DR, reflected on the thinning of the inner retina due to ganglion cell apoptosis with GCL and RNFL thickness decrease, even before microvascular lesions are evident^20,23,35^. The observation that the inner layer thickness further decreased in vitrectomized eyes stresses the need of a special care during the treatment of DME in these eyes.

This was in line with our results. Better visual outcomes were correlated with a lower baseline RNFL, GCL, IPL and INL thickness in central and inner ring ETDRS areas, in both vitrectomized and non-vitrectomized eyes. Moreover, a higher decrease in RNFL inner ring during the 12 months of follow-up was also correlated with a better final vision, in both groups. However, correlations were by far stronger in vitrectomized eyes. These data favors a higher fragility of those eyes when DME does not regress, highlighting the particular nefarious effect of edema in vitrectomized eyes, particularly in the inner ring RNFL, where the axons responsible for central VA are located.

Even though these effects on retinal layer thickness have already been described for non-vitrectomized eyes with DME treated with RBZ or triamcinolone^25^, no prior studies have addressed that effect in vitrectomized eyes. Our study reinforces the need of an optimized DME therapy, especially in non-responder cases and particularly in vitrectomized eyes with a more limited neuroretina reservoir. Although both groups have started with similar BCVA, in vitrectomized eyes the functional outcome was lower, with poor vision in a higher percentage of cases at the end of follow-up, in comparison with non-vitrectomized eyes (50% vs 13.9%, *p* = 0.027).

Another important result was the significant association between good-earlier responders and a lower INL thickness at baseline and at the end of follow-up, especially in the inner ring of ETDRS subfield (*p* = 0.002 and *p* = 0.004, respectively). This may be an important biomarker to predict earlier responders with less treatment burden and a more benign evolution, and may also contribute to the clarification of RD pathophysiology. As it has been described, focal vessel dilations and microaneurisms are among the preclinical vascular changes in DR at the level of the deep capillary layer (DCL) which is located at the INL^36^. According to previous histopathology and OCT studies, microaneurysms are preferentially located at the DCL^37,38^, and a positive correlation has been described between an increased retinal volume and the number of microaneurysms^37^.

The onset of edema in the INL can be considered a consequence of the natural evolution of DCL DR vascular lesions and a sign, when isolated and low grade, of a non-chronic DME, with higher probability to behave as a good-earlier responder to anti-VEGF therapy. Hence, the invasion of adjacent layers can be considered a sign of progression, a higher level of severity and chronicity, particularly when DCL and OPL disruption is evident, characteristics that have been described as predictors of poor response to anti-VEGF and more advanced DR stages, with intra-retinal cysts invading also the ONL, further compromising visual function^39^. From 12 months follow-up to baseline a difference between groups was observed only for INL and, in a lesser extent, for GCL, favoring group 2. The easy access of ranibizumab to the superficial and deep capillary plexus, located in GCL and INL, respectively, through the thinner inner layers which features vitrectomized eyes^32^, may facilitate the stabilization of capillary plexus of those eyes in comparison with non vitrectomized eyes.

CFT may be considered a predictor of early remission of DME under PRN RZB IV injections, as already described^40^. In our study we have also observed a positive correlation between baseline CFT and CFT evolution during the follow-up period, for both vitrectomized and non-vitrectomized eyes. According to our results, 69% of good-earlier responders had a baseline CFT < 400 µm.

In conclusion, vitrectomized and non-vitrectomized eyes achieved an equivalent anatomical outcome and needed a similar number of RBZ IV injections in a PRN regimen. Lower baseline CFT and lower INL thickness seem to be associated to a good-earlier response to RBZ IV therapy. Moreover, the expected compromised inner retina layers thickness in vitrectomized eyes showed to be relevant to their lower functional outcome. Therefore, an optimal treatment choice in order to resolve DME, should be considered promptly to avoid irreversible damages, particularly in those eyes. The severity of the edema compromising the inner retinal layers, in central and inner ring ETDRS areas and its correlation with poor functional outcomes highlight the negative effect of DME to retinal function, especially in those locations.

The results of this study have practical implications when planning schedules for anti-VEGF IV injections, such as loading dose strategy and overall treatment decisions regarding DME approach. Even though the low number of vitrectomized eyes included in this study is a reflection of the relatively infrequent post-vitrectomy DME occurrence^15^, future studies with larger samples should be carried out to further investigate and confirm our results.

## Methods

### Study design

This was a two-center, prospective, observational study, conducted at the Departments of Ophthalmology from Centro Hospitalar e Universitário do Porto (CHUP) and Hospital Santa Maria Maior de Barcelos, Portugal. Fifty consecutive eyes with DME were considered for treatment with RBZ IV injections following a PRN regimen, of which 46 completed the 12 month follow-up. Patients were included in two groups according to the vitreous status: group 1—non-vitrectomized eyes (n = 36); group 2—vitrectomized eyes (n = 10). Patients were followed-up according to the standard of care and a final analysis of the results was conducted at 12 months of follow-up, which was the minimum follow-up period required for each patient. The recruitment period was from January 2018 to January 2019.

This study was conducted according to the tenets of the Declaration of Helsinki in its latest amendment (Brazil, 2013) and was approved by the ethics committee of CHUP [2017.093 (084-DEFI/082-CES)]. All patients signed an informed consent form. This study is registered at www.clinicaltrials.gov (NCT04387604, date of registration 14/05/2020).

#### Inclusion criteria

(1) ≥ 18 years with either type 1 or type 2 diabetes mellitus; (2) central subfield foveal thickness (CFT) > 300 μm, measured using spectral domain optical coherence tomography (SD-OCT, Spectralis HRA + OCT, version 1.10.2.0, Heidelberg Engineering, Heidelberg, Germany); (3) best corrected visual acuity (BCVA) of 20 to 80 letters, using Early Treatment of Diabetic Retinopathy Study (ETDRS) letters chart; (4) minimum period of 6 months post-vitrectomy for inclusion in group 2; (5) ability and willingness to provide written informed consent. (6) if the inclusion critria were fullfilled for both eyes bilateral inclusion was allowed.

#### Exclusion criteria

(1) Epiretinal membrane (ERM) existence in the study eye; (2) persistent posterior hyaloid adherence after vitrectomy for group 2; (3) previous vitrectomy for group 1; (4) history of other retinal vascular diseases in the study eye; (5) LASER photocoagulation or anti-VEGF IV or systemic anti-VEGF or pro-anti-VEGF treatment and cataract surgery in the 6 months prior to study inclusion; (6) IV or peribulbar corticosteroid injections in the 6 months prior to study inclusion; (7) history of IV of implant of fluocinolone acetonide in the study eye; (8) vitreous hemorrhage or opacification in the study eye; (9) proliferative diabetic retinopathy (PDR) in the study eye; (10) active ocular inflammation or infection in either eye; (11) aphakia in the study eye; (12) other causes for macular edema, e.g., after cataract surgery in the study eye; (13) other causes of visual loss in the study eye; (14) pro-edematous medication (such as systemic glitazones or prostaglandins) or other pathologies that might influence the course of macular edema in the study eye; (15) uncontrolled glaucoma in either eye (intraocular pressure > 24 mmHg with treatment); (16) history of stroke in the previous 6 months; (17) uncontrolled arterial hypertension (systolic blood pressure > 160 mmHg or diastolic blood pressure > 100 mmHg).

### Treatment

All patients were treated with RBZ IV injections (0.5 mg/0.05 ml) following a PRN regimen. In the PRN regimen adopted, the rationale was to follow the patient every 4 weeks and treat every 4 weeks until a maximum BCVA (85 letters) without edema or stability were achieved. Stability was considered when CFT was < 300 mm or when a change in BCVA of < 5 letters or CFT < 10% in two consecutive visits within the 24 week period, considered a critical period of therapy, were observed.

When required, adjunct treatment with macular LASER (rescue LASER) was also admitted at or after 24 weeks, in case of persistent DME.

### Treatment schedule

**Repeat injections** every 4-weeks if eye “improved” or “worsened” (defined as ≥ 5 letter change from last injection, or ≥ 10% CFT increase on OCT from last injection), or if CFT > 300 μm at any time point. BCVA worsening was only considered a treatment criterion if it was due to DME and not with other ocular cause.

**Defer injections** if either BCVA of 85 letters and OCT CFT was “normal” (CFT ≤ 300 μm and non-existent intra- or sub-retinal fluid); or OCT CFT was “normal” (CFT ≤ 300 μm) and stable BCVA (defined as < 5 letters change from last injection) after two consecutive injections during the first 24 weeks, or after one injection if OCT and VA criteria were equal or better than those obtained in a previous stability period.

### Patient assessment

At baseline, demographic and clinical data, including, serum levels of hemoglobin and glycated hemoglobin (HbA1C), microalbuminuria, body mass index and systolic and diastolic blood pressure were recorded. Each patient performed an SD-OCT, and underwent a complete ophthalmological evaluation (symptoms, BCVA, intraocular pressure measurement, anterior and posterior segment biomicroscopy) at baseline and every month until the end of follow-up. Fluorescein angiography was performed on all patients at study entrance and at the end of follow-up.

### SD-OCT acquisition, data collection and data assessment

Two highly trained technicians conducted SD-OCT scans. Macular thickness measurements were performed with automated segmentation, 25 scans and a 20° × 20° acquisition mode. The follow-up function and auto-rescan with active eye tracking were also utilized. CFT was obtained automatically from equipment readings.

The thickness of seven retinal layers—retinal nerve fibre layer (RNFL), ganglion cell layer (GCL), inner plexiform layer (IPL), inner nuclear layer (INL), outer plexiform layer (OPL), outer nuclear layer (ONL) and outer retinal layer (ORL)—between the external limiting membrane and the bruch membrane—were also measured. Automatic segmentation errors were corrected manually when necessary.

**Figure 3:**
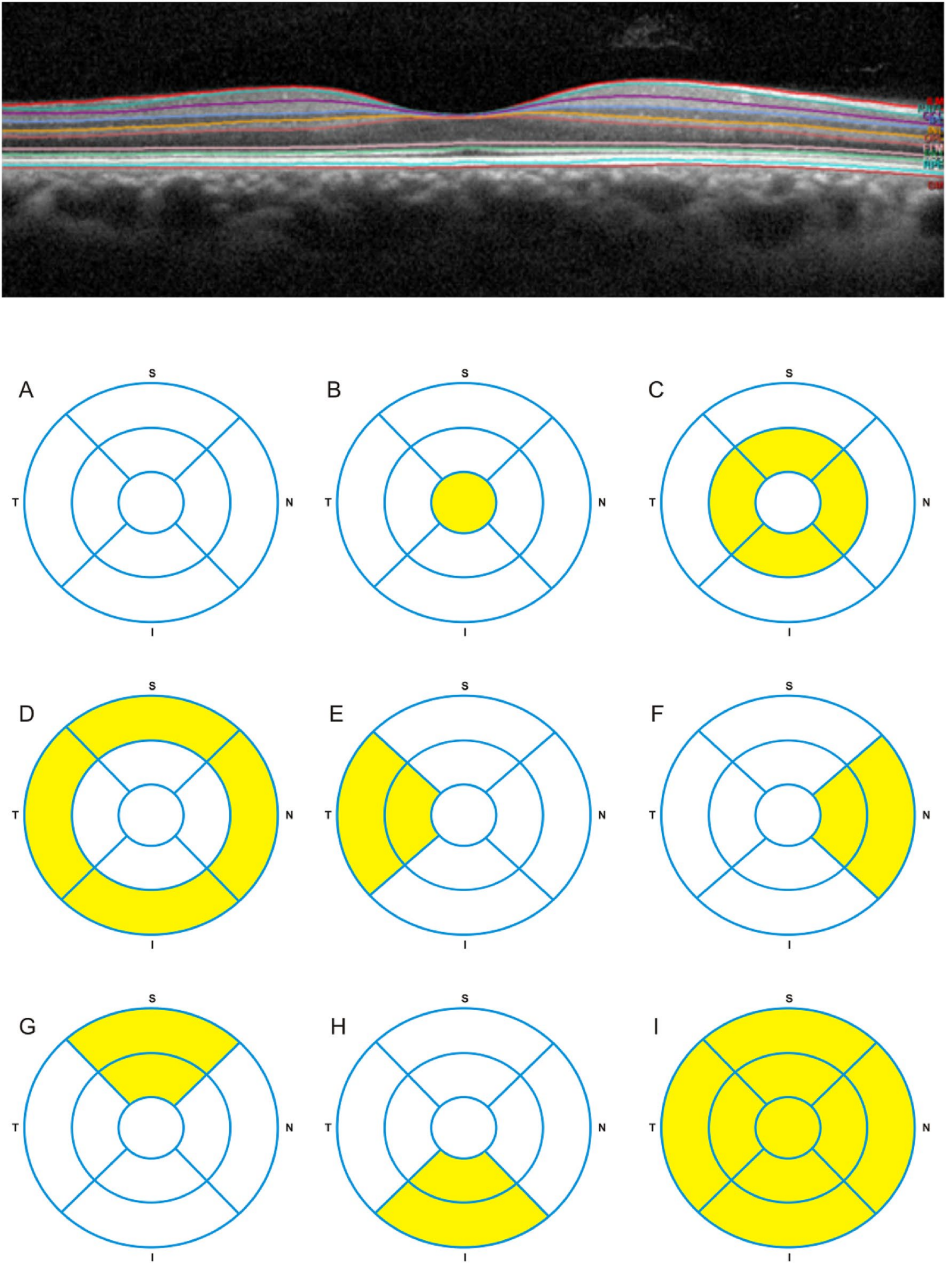
Retinal layers were identified by automatic segmentation in SD-OCT—retinal nerve fibre layer (RNFL), ganglion cell layer (GCL), inner plexiform layer (IPL), inner nuclear layer (INL), outer plexiform layer (OPL), outer nuclear layer (ONL) and outer retinal layer (ORL)—between the external limiting membrane and the bruch membrane (top panel). The mean thickness of each individual retinal layer was analysed in: (1) the nine individual ETDRS subfields (**A**); the fovea (or central circle with a diameter of 1 mm) (**B**); the inner ring ETDRS subfields (**C**); the outer ring ETDRS subfields (**D**); and globally (**I**). For RNFL and GCL the mean layer thicknesses of the outer and inner temporal (**E**), nasal (**F**), superior (**G**) and inferior (**H**) (bottom panel). Bottom panel adapted from Won et al.^32^.

The mean thickness of each individual retinal layer was analysed in: (1) the nine individual ETDRS subfields; (2) the inner ring ETDRS subfields; (3) and the outer ring ETDRS subfields. For RNFL and GCL the mean layer thicknesses of the inner and outer nasal, temporal, superior and inferior ETDRS subfields were also calculated. Figure 3 shows a representative image of the retinal layers identified by automatic segmentation in SD-OCT scan and a schematic representation of the nine individual ETDRS subfields, this latter adapted from Won et al.^32^.

### Outcome measures

#### Primary outcome

To assess the differences between groups in the number of RBZ IV injections needed to control DME.

#### Secondary outcomes

Secondary endpoints included: (1) comparison of groups (1.1) BCVA and CFT at baseline and after 12 months of follow-up; (1.2) differences in type of responders; (1.3) analysis of the thickness of seven retinal layers; (1.4) correlation between retinal layers thickness and BCVA; (2) safety.

#### Functional and anatomical outcomes criteria

A significant functional improvement was defined as a gain ≥ 5 ETDRS letters. A DME resolution was defined as CFT ≤ 300 µm. BCVA ≥ 70 letters was defined as a good visual acuity and BCVA < 70 as a poor visual acuity. Type of responder was classified as: (1) good-earlier responder—when beyond the 24th week of follow-up (maximum 7 injections) there was a complete anatomical response (CFT ≤ 300 μm) with an increase in BCVA ≥ 5 letters; (2) non-responder—13 injections and final CFT > 400 µm or ≤ 10% of CFT reduction and BCVA gain < 5 letters; (3) partial responder—between good-earlier responder and non-responder criteria. According to our definition, a late responder was considered a partial responder.

#### Statistical analysis

Data were analyzed using non-parametric statistics. BCVA values in ETDRS were converted to LogMar before analyses. Between-group analyses of continuous variables were performed using the Mann–Whitney U test. Within-group analyses were performed using the Wilcoxon test. Nominal variables were analyzed using the chi-square test. Correlations between variables were tested using the Spearman rank correlation or the Kendall’s τ−b, as appropriate. A *p* < 0.05 (two-sided) was considered statistically significant.

## Data Availability

The datasets generated during and/or analysed during the current study are available from the corresponding author on reasonable request.
